# Transstyloid, transscaphoid, transtriquetral perilunate dislocation - A late presentation

**DOI:** 10.4103/0019-5413.65147

**Published:** 2010

**Authors:** Shiju A Majeed, S Manoj Kumar

**Affiliations:** Department of Orthopedics, Govt. Medical College, Trivandrum, Kerala, India

**Keywords:** Carpal dislocations, Joshi’s distraction system, ligamentotaxis, perilunate dislocation

## Abstract

Transstyloid, transscaphoid, transtriquetral perilunate dislocations are extremely rare carpal dislocations. We report a 24-year-old male who presented with this rare injury pattern four weeks after sustaining trauma. The patient underwent open reduction and internal fixation via dorsal approach. Reduction was assisted by the use of Joshi’s Distraction System. Scaphoid fracture healed by 16 weeks. At 2 years follow-up patient has good range of motion around wrist without any discomfort.

## INTRODUCTION

Transstyloid, transscaphoid, transtriquetral perilunate dislocations are extremely rare injuries. They involve the greater arc of wrist joint; 25% of perilunate dislocations are missed at the initial presentation. There are not many reports of such an injury in the literature. We report such a case which presented 1 month after trauma.

## CASE REPORT

**Figure 1:**
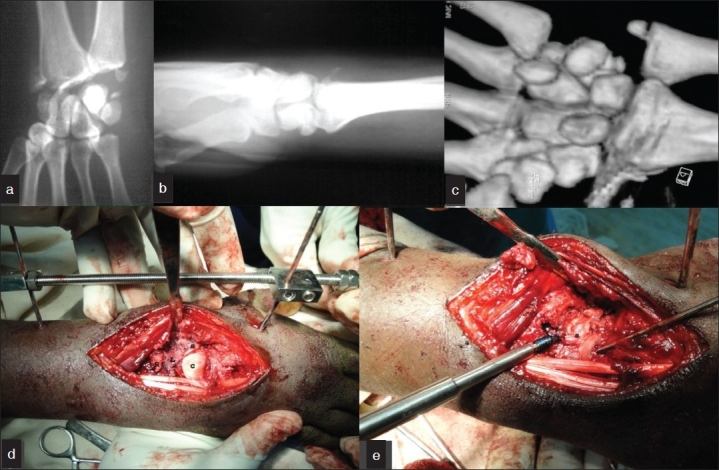
(a) Radiograph (anteroposterior view) showing the transstyloid, transscaphoid, transtriquetral perilunate dislocation. (b) Lateral view showing carpal dislocation. (c) CT scan showing loss of congruity of the articular surfaces between the proximal arc and distal arc of the wrist. Fracture line through the waist of scaphoid fracture across the triquetrum, and intraarticular fracture across the radial styloid. Capitate is seen displaced dorsally and proximally abutting the radius. Intraoperative photograph showing (d) the dislocated capitate. (e) The reduction of the carpus after ligamentotaxis (C = capitate, S = scaphord, R = radius, L = lunate)

A 24-year-old male presented with pain and swelling in his right wrist one month after fall from bike. He underwent Ayurvedic treatment prior to presentation. On examination, his right wrist was swollen with abnormal prominence on the dorsal aspect. The wrist was tender with gross limitation of all movements. Neurovascular examination was normal. His radiographs showed fractures of both radial and ulnar styloids, fracture of scaphoid at the waist and chip fracture of triquetrum. The capitate and distal scaphoid had displaced dorsally. The capitate was overlying the lunate and was found to abutt the distal radius. A diagnosis of transstyloid, transscaphoid, transtriquetral perilunate dislocation was made [Figure 1a‐c].

**Figure 2:**
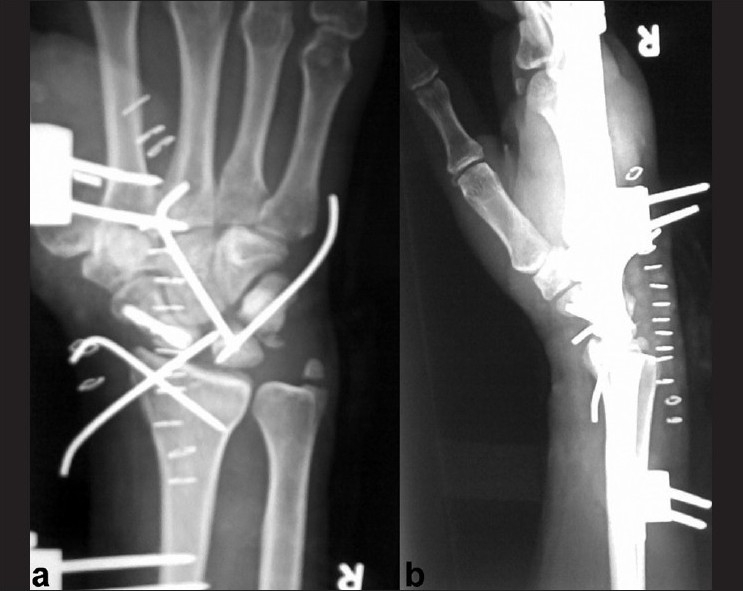
Postoperative anteroposterior (a) and lateral (b) radiograph showing good carpal alignment

**Figure 3:**
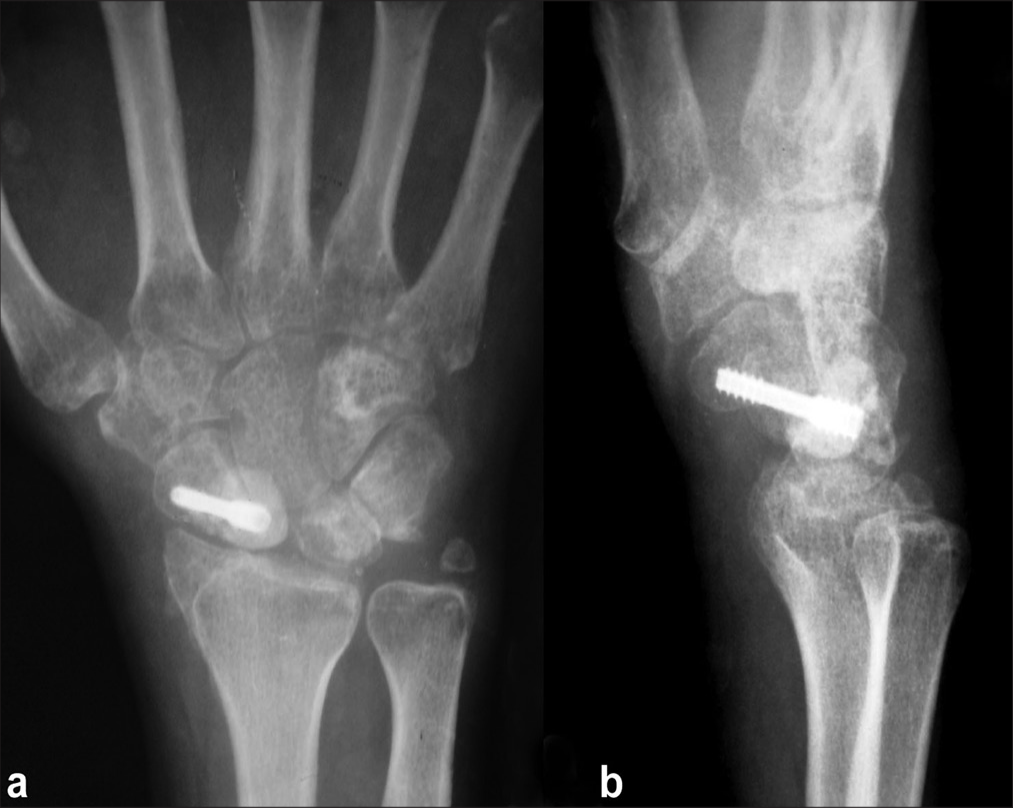
Follow-up anteroposterior (a) and lateral (b) radiograph showing the healed scaphoid fracture. The carpal bones are in acceptable alignment

**Figure 4:**
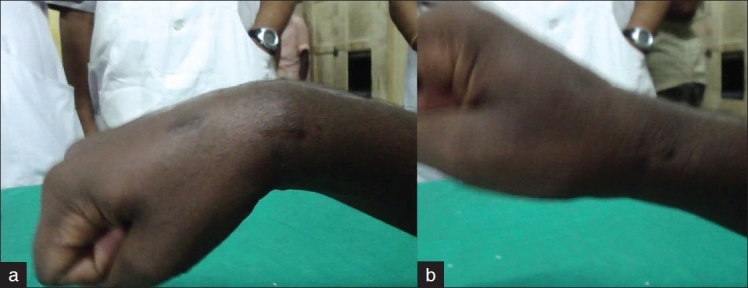
(a, b) Clinical photograph showing grip and range of motion at wrist

The patient underwent open reduction of the fracture dislocation under supraclavicular block. Dorsal approach to the wrist joint over the tendon of extensor pollicis longus was used. The dorsal wrist capsule was incised in a lateral based V fashion so as to least injure the dorsal radiocapitate ligaments and dorsal intercarpal ligaments.^1^ Radial styloid was fixed first with K wire. Joshi’s distractor was applied after placing two 3.5mm Schanz pins in the radius and two 2.5mm Schanz pins in the second metacarpal in the standard fashion. The wrist joint was distracted between the two sets of Schanz pins employing the principle of ligamentotaxis. This helped to bring the capitate in alignment with the lunate. The Scaphoid was reduced and fixed with a Herbert screw. Scapho lunate ligament was found to be intact. Triquetral fracture was minimally displaced but involving the lunotriquetral articular surfaces. K wire was used to transfix triquetrum to lunate after denuding the adjoining cartilage. Capitolunate transfixing K wire was inserted. The intraoperative photographs are shown [Figure 1d and e]. Reductions were checked under C arm [Figure 2a and b]. The dorsal capsule was closed meticulously and skin, subcutaneous tissues approximated. The distraction of the external fixator was completely reduced and checked with C arm. The fixator was left in place until suture removal to help in easier wound inspection and early institution of physiotherapy. Patient was encouraged to mobilize the fingers. After suture removal, a short arm cast was applied. K wires were left for six weeks. On removal of K wires, patient was placed on physiotherapy. Scaphoid fracture healed by 16 weeks [Figure 3a and b]. On two-year follow-up, patient has 70 degrees of palmar flexion, 50 degrees of dorsiflexion and is able to perform most activities without discomfort [Figure 4a and b].

## DISCUSSION

Gellman *et al*.,^2^ reported on a late presenting dorsal transscaphoid, transtriquetral, perilunate wrist dislocation with open reduction and K wire fixation. But this patient did not have transstyloid element. Campbell 3 reported on a transcaphoid dorsal perilunate dislocation with styloid fracture. Wesley and Barenfield 4 reported on an acute case of transscaphoid, transcapitate, transtriquetral perilunate fracture dislocation of the wrist. Here again the radial styloids were intact. Stevanovic *et al*, 5 reported on an acute case of transscaphoid, transtriquetral, volar lunate fracture dislocation of the wrist. Grissis *et al*, 6 described a neglected transscaphoid, transstyloid, volar dislocation of lunate which presented after six weeks post injury. They did open reduction and fixation with K wire using combined dorsal and volar approach. A satisfactory result could be obtained.

Many experts 7–9 believe that open reduction to achieve good carpal alignment gives the best outcome in perilunate dislocations even when they present late. The traditional way to approach carpal dislocations which present late is to use combined dorsal and volar approach to the wrist joint. This often needs division of the dorsal and palmar wrist ligaments. Palmar ligaments are more important in the stability of the wrist joints and hand grip strength. So it becomes imperative to meticulously repair the ligaments. In patients who undergo open reduction six to eight weeks after the injury, these ligaments are so attenuated to make ligament repair difficult. The use of external fixator with distraction along with single dorsal approach helps to preserve the palmar wrist ligaments to the maximum. There is minimal surgical soft tissue damage. This helps in earlier functional return of the patient. If satisfactory reduction is not possible, such dislocations can be treated by the options of proximal row carpectomy, or wrist arthrodesis. The soft tissue damage coupled with late treatment has the potential risk of avascular necrosis of scaphoid and lunate. However, radiographic opacities alone do not compromise a good outcome. If the fracture goes to union, there is a good chance for revascularization through the healed fracture site and also through the intact scapholunate ligaments.

Carpal dislocations are often missed injuries. Prompt identification and care is required for optimal outcome. Herzberg and associates in a multicentre study reviewed the outcome of 115 perilunate injuries with a mean six-year follow-up. They noted that although the pattern of injury had little influence on outcome, delay in treatment did adversely affect results. The best results were achieved with open reduction and internal fixation. Careful clinical examination with attention to the history is required to prevent missing these injuries. Careful scrutiny of radiographs is essential. One has to pay attention to the Gilula’s arc of the carpal joints, scapholunate interval, scaphocapitate and capitolunate angles to diagnose subtle wrist injuries. CT with 3D reconstruction is useful in better outlining the problem. During surgery in late cases, lot of scar tissue is seen between the capitate and lunate which makes alignment of the carpus difficult. This can result in unnecessary severing of many important ligaments. Applying distraction using the external fixator helps in proper and easy alignment of carpus under vision with optimal preservation of ligaments. Fractured Proximal pole of scaphoid and lunate are often seen as a single piece and the distal scaphoid fragment is usually displaced dorsally.

In summary, with good cartilage, open reduction should be attempted even in late cases of such carpal dislocations. External fixator with controlled distraction is a useful surgical tool in obtaining reduction with minimum ligament strippage.
